# The Medical Care of Children in Durham

**Published:** 1896-06-20

**Authors:** 


### The Medical Care of Children in Durham.

The Society for the Prevention of Cruelty to Children must surely be unacquainted with the condition of things in regard to the medical care of children in the county of Durham. It and the public should alike consider some striking facts in this connection made public by the medical officer of health for the county, Dr. Eustace Hill. It is recognised that to frequently underfeed a child is cruelty, and cruelty which the law takes cognisance of and punishes. It is cruelty also to beat a child unmercifully. But we venture to think that neither of those forms of cruelty is so seriously "cruel" as the depriving of a child in mortal sickness of competent medical aid. For the two reasons of greater immediate suffering and of a much higher degree of danger to health and life, the child untended by skilled hands in serious illness is, or should be, an object of much more sympathy than the child who is severely beaten or occasionally underfed. This is a view which a careful study of the subject will establish in any reasoning person's mind, but it is a view, we venture to affirm, which is hardly ever present for practical purposes in any mind except the medical.

In certain portions of the county of Durham the systematic deprivation of young children in the matter of skilled medical aid, if we may trust Dr. Eustace Hill, has reached a pitch which is shamefully wicked. The magnitude of this most inhuman evil may be guessed at by considering the proportion of "uncertified" to "certified" deaths among those children who succumb to mortal illnesses. For it is pretty certain that whatever be the proportion of children suffering from deadly diseases who are denied skilled medical attendance, the proportion will be still larger among those children who suffer from diseases which fall short of being "deadly." Dr. Eustace Hill tells us that in those parts of Durham where this evil has reached its greatest magnitude more than half of the children whose deaths are registered die without skilled medical attendance. To be strictly precise, of children under one year old who died last year, the deaths of 55·7 per cent. were "uncertified"; whilst of children between the ages of one and five years the deaths of 72·1 per cent. were uncertificated. Even in the case of those dying from zymotic diseases the proportion of "uncertified" to "certified" deaths was as 16·0 to 12·07.

Now, as we have said, and as every practising doctor knows quite well, an uncertified death is the death of a person who in life, or, at any rate, during his mortal illness, has received no competent medical aid. Here, then, we have in parts of the county of Durham far more than half the children who are "down" with mortal diseases entirely denied the advantages of such skilled medical aid as would certainly reduce their sufferings to a minimum, and as would, in many cases, undoubtedly save them from an untimely death. There is no doubt about the facts, and they are of a most distressing, shocking, and shameful kind; and if, without any sensational embellishment whatever, they could be thrown upon the screen of the public imagination for a very few weeks, a revolution would, we think, be effected in the conditions of the medical care of children which would sweep away all such inhumanity as we have described at once and for ever.

If we are asked what of a practical kind can be done? the answer is, stamp out unqualified medical practice, at once and for ever. In every department of life the public is demanding that all people who are entrusted with responsible work should give some assurance that they have been properly trained, and that they are qualified for the performance of their duties. Schoolmasters, postmen, even cabmen, undergo examination. But in medicine, any man fresh from the loom or the anvil is thought good enough. And so long as this is allowed to continue the children will suffer and die.

